# *N*-Methyl-D-aspartic Acid (NMDA) in the nervous system of the amphioxus *Branchiostoma lanceolatum*

**DOI:** 10.1186/1471-2202-8-109

**Published:** 2007-12-20

**Authors:** Salvatore D'Aniello, George H Fisher, Enza Topo, Gabriele Ferrandino, Jordi Garcia-Fernàndez, Antimo D'Aniello

**Affiliations:** 1 Departament de Genètica, Facultat de Biologia, Universitat de Barcelona, Avinguda Diagonal 645, 08028 Barcelona, Spain; 2 Department of Chemistry, Barry University, Miami Shores, Florida 33161, USA; 3 Laboratory of Neurobiology, Stazione Zoologica "A. Dohrn", Villa Comunale 1, 80121 Napoli, Italy

## Abstract

**Background:**

NMDA (*N*-methyl-D-aspartic acid) is a widely known agonist for a class of glutamate receptors, the NMDA type. Synthetic NMDA elicits very strong activity for the induction of hypothalamic factors and hypophyseal hormones in mammals. Moreover, endogenous NMDA has been found in rat, where it has a role in the induction of GnRH (Gonadotropin Releasing Hormone) in the hypothalamus, and of LH (Luteinizing Hormone) and PRL (Prolactin) in the pituitary gland.

**Results:**

In this study we show evidence for the occurrence of endogenous NMDA in the amphioxus *Branchiostoma lanceolatum*. A relatively high concentration of NMDA occurs in the nervous system of this species (3.08 ± 0.37 nmol/g tissue in the nerve cord and 10.52 ± 1.41 nmol/g tissue in the cephalic vesicle). As in rat, in amphioxus NMDA is also biosynthesized from D-aspartic acid (D-Asp) by a NMDA synthase (also called D-aspartate methyl transferase).

**Conclusion:**

Given the simplicity of the amphioxus nervous and endocrine systems compared to mammalian, the discovery of NMDA in this protochordate is important to gain insights into the role of endogenous NMDA in the nervous and endocrine systems of metazoans and particularly in the chordate lineage.

## Background

**Figure 1:**
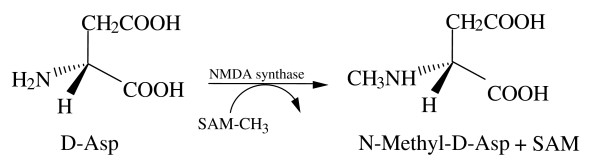
D-Aspartic acid is transformed in NMDA by substitution of a H^+ ^atom of the amino group (NH_2_) with a methyl group (CH_3_).

Synthetic *N*-methyl-D-aspartic acid (NMDA) is widely known for its property to be an agonist of the L-glutamate receptor of the NMDA-type. Among the three families of glutamate receptor proteins identified, one is activated by NMDA. The subunits of this receptor (NR1, NR2A, NR2B, NR2C and NR2D of the ligand-gated ionic channels) are collectively referred to as glutamate receptors of the NMDA-type. Another receptor family, known as AMPA-type, is activated by alpha-**a**mino-3-hydroxy-5-**m**ethyl-4-isoxazole **p**ropionic **a**cid (AMPA) and by kainate. The third family of glutamate receptors consists of G-protein coupled receptors, the so-called matabotropic receptors (mGluR1-8) [1,2] for its widespread activity, among which one of the most important is to induce hormone release in the hypothalamus and pituitary gland [3-6]. NMDA was first isolated as endogenous compound in the marine mollusk *Scapharca brougtonii *in 1987, by cation exchange chromatography [7], and later by HPLC methods [8]. Recently, using a novel strategy consisting in the purification of the tissue sample with *o*-phthaldialdehyde (OPA) associated with an enzymatic HPLC method, we demonstrated that NMDA is present in neuroendocrine tissues of rat [9,10], of the protochordate *Ciona intestinalis *[11] and in other animal phyla [12]. In addition, it has been also demonstrated that endogenous NMDA is a molecule derived from D-aspartic acid (D-Asp), in which an hydrogen atom of the amino group (NH_2_) at the alpha carbon position of D-Asp is substituted by a methyl group (CH_3_) by a NMDA synthase (also called: D-aspartate methyl transferase), according to the reaction in Figure 1[9-11].

Amphioxus is an emerging animal model, situated in a key phylogenetic position, as a descent of a chordate ancestor. With vertebrates, amphioxus shares many body plan characteristics such as a dorsal nerve cord, notochord, endostyle, segmental muscles, pharyngeal gill slits and a post-anal tail. The genetic programs patterning the amphioxus embryo are also similar to those of vertebrate embryos, even if they are supported from a limited set of genes. In fact, cephalochordates did not undergo the extensive genome duplications that gave raise to more complex pathways and the gene redundancy typical of polyploid organisms, such as vertebrates.

Noticeably, in the last few years the position of cephalochordates and tunicates has been reversed. In fact, an accurate phylogenetic analysis of a large amount of molecular data indicated that urochordates are the closest relatives to vertebrates, while cephalochordates branched earlier [13]. This change in the chordate phylogeny has profound implications for the understanding of the evolution of body plans, and it may imply that the chordate ancestor had a body plan organization similar to contemporary amphioxus.

Taking this into consideration, cephalochordates represent a valuable model organism in understanding chordate's evolution, and in particular to distinguish between conserved (potentially ancestral) and derived (potentially novel) characteristics of vertebrates.

Recently, we reported that the amphioxus nervous system contains considerable amounts of free D-aspartic acid [14]. Since in rat and in Ciona this amino acid constitutes the precursor for the *in vivo *biosynthesis of NMDA [9-11], in the present study we have designed approaches to reveal if NMDA is also present in the amphioxus nervous system and to know if this excitatory amino acid is biosynthesized from D-aspartic acid.

## Results

### Purification of NMDA and HPLC determination

**Figure 2:**
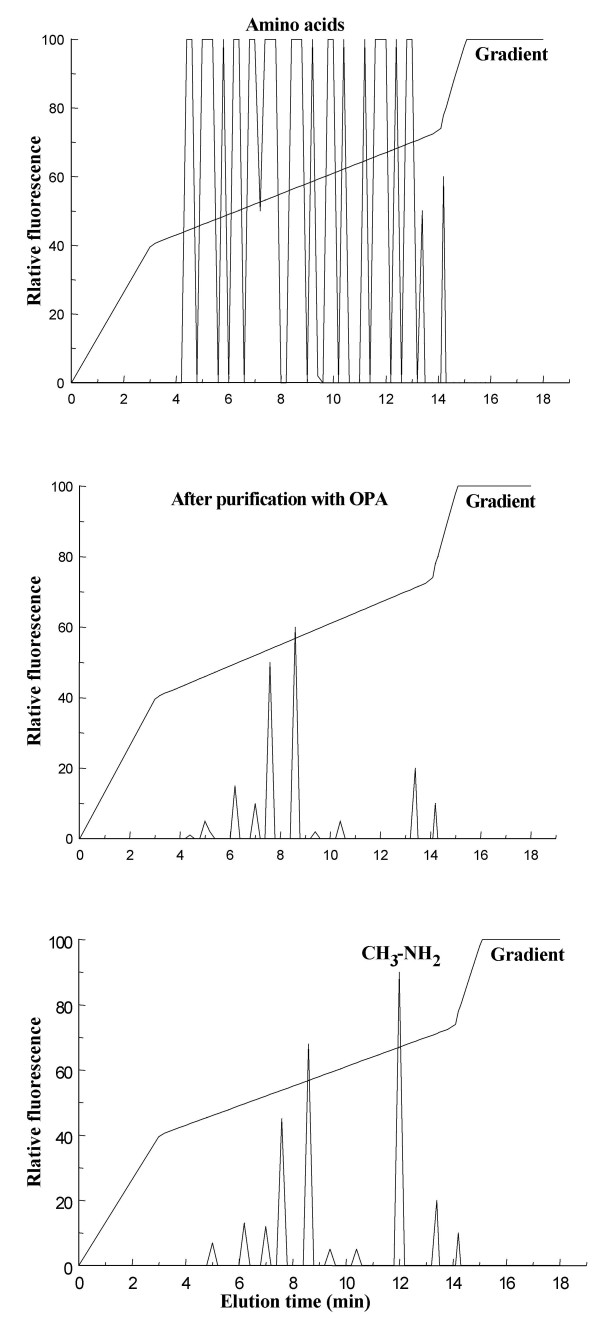
**HPLC analysis of NMDA through the determination of CH_3_NH_2 _of the nerve cord after purification with OPA**. **A) **The figure shows a typical pattern of free amino acids from *B. lanceolatum *nerve cord before purification by OPA treatment. **B) **The same sample after purification with OPA, which eliminates all the amino acids (or almost all) except NMDA. Note that it is not possible to see the NMDA in this graphic because it does not react with OPA-mercaptoethanol, that is the reagent used for the determination of free amino acids at HPLC. **C) **The same sample as B, but after treatment with D-AspO. In this case, the D-AspO oxidizes NMDA producing the CH_3_NH_2 _which reacts with OPA-mercaptoethanol to give a well-defined sharp peak at the end of the chromatogram at retention time 11.8–12.0 min.

To determine specifically NMDA contained in an amphioxus homogenate, the sample was highly purified from other amino acids and amino compounds, then the NMDA content in the purified sample was determined by HPLC. The purification of NMDA from a crude homogenate was an essential step in order to avoid interference by the chromatographic peaks of other amino acids that could overlap with the peak of methylamine, CH_3_NH_2 _(generated from the oxidation of NMDA with D-AspO). In fact, after the sample has been purified with OPA, almost 95–98% of all NMDA is purified from other compounds. The use of the D-aspartate oxidase for the determination of NMDA was another essential condition, because NMDA as such does not react with OPA-mercaptoethanol to form a fluorescent complex. Therefore, NMDA was first oxidized with D-AspO to form CH_3_NH_2 _and then treated with OPA-mercaptoethanol. In this way the CH_3_NH_2 _reacts with OPA-mercaptoethanol giving a fluorescent chromatographic peak. Figure 2 shows a typical example of NMDA determination from a pool of amphioxus nerve cords. In panel A, an analysis of total amino acids obtained from 2 mg of tissues is shown. Panel B shows the same sample after OPA purification, in this case almost all the amino acid peaks have disappeared. Furthermore, when the sample is treated with D-AspO there is an increase of the peak at elution time of about 12 min due to the formation of CH_3_NH_2 _derived from the oxidation of NMDA (Fig. 2, panel C).

### Comparative concentration of NMDA between amphioxus and other animal phyla

In this study, we have improved the previously described procedure for the purification of NMDA from biological samples [9,10], showing that purified NMDA from the nervous system of the amphioxus *B. lanceolatum *(Table 1) can be obtained with minimal loss during the procedure.

**Table 1:** Concentration of *N*-Methyl-D-Aspartic acid (NMDA) in the nervous system of amphioxus *Branchiostoma lanceolatum*.

	Nerve Cord	Cephalic Vesicle/Hindbrain	Muscle
	nmol/g wet tissue		
	
1^st ^Pool	2.8	10.2	0.62
2^nd ^Pool	3.1	9.4	0.51
3^rd ^Pool	3.7	12.7	0.55
4^th ^Pool	3.2	8.8	0.59
5^th ^Pool	2.6	11.5	0.48

Mean ± S.D.	3.08 ± 0.37	10.52 ± 1.41	0.55 ± 0.051

* Each pool of tissue was obtained from 20 adult amphioxus.

Table 2 compares the concentration of NMDA found in the amphioxus CNS with nervous tissues from different animal phyla, such as chordates: mammals (*Rattus norvegicus*), birds (*Gallus gallus*), amphibians (*Rana esculenta*), ray fish (*Torpedo ocellata*) and tunicates (*Ciona intestinalis*); arthropods: crustacean (*Carcinus maenas*); and mollusks: cephalopod (*Octopus vulgaris*).

**Table 2:** Comparative study on the occurrence of NMDA in the nervous system of various animal species.

**Phyla**	**Tissues**	**nmol/g tissue**
**Chordates**		
Mammals (*Rattus norvegicus*)^#^	brain	1.40 ± 0.30
Birds (*Gallus gallus*)*	brain	2.20 ± 0.20
Amphibians (*Rana esculenta*)*	brain	2.24 ± 0.38
Fishes (*Torpedo ocellata*)*	brain	7.76 ± 0.85
Tunicates (*Ciona intestinalis*)*	cerebral ganglion	13.8 ± 2.65
		
Cephalochordates	nerve cord	3.08 ± 0.37
(*Branchiostoma lanceolatum*)°	cephalic vesicle	10.52 ± 1.41
		
**Arthropods**		
Crustaceans (*Carcinus maenas*)*	brain	4.46 ± 0.73
		
**Mollusks**		
Cephalopod (*Octopus vulgaris*)*	optic lobes	2.20 ± 0.48

^# ^from D'Aniello *et al*., 2000 [7]; * from D'Aniello *et al*., 2002 [10]; ° Present work.

The concentration of NMDA in the nervous tissue of several animals ranges normally between 1.4 and 7.6 nmol/g of tissue, except for the cerebral ganglion of *Ciona intestinalis*, where NMDA occurs at considerably higher concentrations, 13.8 nmol/g of tissue (Table 2). Interestingly also the amphioxus brain contains a very high NMDA concentration (10.52 ± 1.41 nmol/g).

### Biosynthesis of NMDA

In rat [7] and in ascidians [9] NMDA is biosynthesized from D-aspartic acid, which represents the precursor amino acid for the synthesis of this molecule, and SAM (*S*-Adenosyl Metionine) as the methyl group donor. In this study we carried out parallel experiments, finding a very high NMDA biosynthetic activity when D-Asp was used as substrate and when we used extracts from the same amphioxus tissues in which NMDA was found endogenously (Table 3). In fact, the nerve cord and particularly the brain were the tissues in which a higher amount of NMDA was synthesized: 33.4 ± 4.30 and 45.5 ± 5.4 nmol/g of tissue, respectively. However, muscular tissues show a poor NMDA synthase activity (1.5 ± 0.4 nmol/g of tissue). It seems, thus, that the amount of endogenous NMDA is proportionally comparable with *in vitro *activity of D-aspartate methyl transferase enzyme, able to transform the D-aspartic residue into NMDA.

**Table 3:** Biosynthesis of NMDA from D-Asp and other amino acids in the nervous tissues of the amphioxus *Branchiostoma lanceolatum*.

Amino acid *	Tissue		
	
	Nerve Cord	Cephalic Vesicle/Hindbrain	Muscle
	NMDA synthesized (nmol/g tissues)		
	
**D-Asp**	**33.4 ± 4.3**	**45.5 ± 5.4**	**1.5 ± 0.4**
L-Asp	3.4 ± 0.4	3.2 ± 0.6	0.8 ± 0.2
D-Glu	3.8 ± 0.5	2.3 ± 0.4	0.7 ± 0.1
L-Glu	3.5 ± 0.4	2.1 ± 0.4	0.6 ± 0.1
D-Ala	3.2 ± 0.3	2.2 ± 0.3	0.5 ± 0.2
L-Ala	3.3 ± 0.4	3.2 ± 0.6	0.8 ± 0.2
D-Ser	2.9 ± 0.4	2.6 ± 0.4	0.6 ± 0.2
L-Ser	3.5 ± 0.6	1.7 ± 0.2	0.7 ± 0.2
Gly	3.6 ± 0.4	1.9 ± 0.5	0.6 ± 0.2
			
Control	3.1 ± 0.4	2.3 ± 0.4	0.5 ± 0.1

* Tissues were incubated with the amino acids listed in the table and SAM, according to the method described in the "Methods" section. Results are the mean ± S.D. obtained from 3 experiments.

In order to know if only D-Asp constitutes the NMDA precursor molecule, several different amino acids, in both D- and L-conformation, were used as potential substrate in the same reaction conditions as previously described for D-Asp (Table 3). The results obtained demonstrate that, except for D-Asp, no other amino acid is able to act as precursor for the synthesis of NMDA.

## Discussion

We recently found that the neural system of amphioxus contains a considerable amount of D-aspartic acid, approximately 280 nmol/g of tissue [14]. In the present work we found in the nervous system of *B. lanceolatum *a considerable quantity of NMDA in different regions (nerve cord and brain) similar to that found in the cerebral ganglion of *Ciona intestinalis *(Tables 1 and 2). In addition, as occurs in rat and in urochordates, NMDA is synthesized also in amphioxus from D-Asp by a SAM-dependent NMDA synthase, which transports a methyl group from SAM to D-Asp, transforming it into NMDA (Table 3). Thus, as in other animals, a biological pathway for the endogenous production of NMDA seems to exist in amphioxus.

Recently, we also demonstrated that NMDA is present in the rat neuroendocrine system, in *C. intestinalis *and in other animal phyla [9-12]. Synthetic NMDA is well known for its stimulating action on the NMDA-type L-glutamate receptors. In addition to having this effect, NMDA is also known for inducing hormone release in the hypothalamus and hypophysis. In fact, already back in 1978, Price and collaborators found that a very low amount of NMDA injected into the rat increased LH levels [3]. After that, Gay and colleagues [5] found that NMD/LA (*N*-methyl-D/L-aspartic acid) elicits hypothalamic gonadotropic release in the Rhesus monkey (*Macaca mulatta*), and Downing and colleagues [6] observed that a minute quantity of NMD/LA injected into the ewe induced the release of gonadotropin releasing hormone (GnRH) from the hypothalamus, and growth hormone (GH) and prolactin (PRL) from the pituitary gland. It has also been demonstrated that in rat synthetic NMDA is able to elicit the release of LH and PRL [9,10].

In addition, an elevation of testosterone and progesterone concentration in rat has been observed as a consequence of the LH increase [9]. The same phenomenon has been shown to occur in *C. intestinalis *[11]. In tunicates, interestingly, D-Asp is present in the cerebral ganglion while NMDA is present in the neural gland. NMDA is synthesized in the neural gland from D-Asp as precursor and in turn it induces the release of the GnRH. Then, GnRH reaches the gonads where it induces the synthesis and release of testosterone and progesterone. Thus, in both mammals and in the protochordate *Ciona intestinalis*, NMDA is involved in hormone production and activity [11]. Although our data do not allow to ascertain the functional effects of NMDA in amphioxus physiology or endocrine system, its high concentration in the brain area is suggestive of it, and indicates that NMDA-driven actions most probably predated chordate evolution.

## Conclusion

The presence of NMDA primarily in the cephalic vesicle of the amphioxus *B. lanceolatum *led us to argue that in cephalochordates NMDA may have a role in both nervous and endocrine systems. Thus, as in mammals and in tunicates, where D-Asp and NMDA are implicated in the development and function of the neuroendocrine system, it is reasonable to suggest that the presence of D-Asp and NMDA in the nervous system of the amphioxus gives support to the concept that modern vertebrates and cephalochordates share a common chordate ancestor, in which D-Asp and NMDA neurotransmission could play a relevant function in the neuroendocrine system. Although data in other deuterostomes (echinoderms and hemichordates) are not available, the presence of NMDA in the nervous system of several mollusks may indicate that NMDA-derived neurotransmission could even be a more general character of Bilateria.

## Methods

### Animals collection and chemicals

Adult amphioxus *Branchiostoma lanceolatum *were collected in the bay of Argelès-sur-Mer (southern coast of France) in January 2007 as described in Fuentes *et al*., 2007 [15]. Adult animals were kept for a week before dissection in glass tanks in the laboratory at a water temperature of 15°C and a 10/14 day-night light cycle (corresponding to natural conditions). Filtered seawater was changed daily and kanamycin antibiotic (10 mg/l sea water) was added to prevent bacterial contamination. Specimens were dissected under an optic microscope using a micro-fine knife with a 4 mm cutting edge (F.S.T.) on ice. No anaesthetic was used to immobilize the animals, in order to avoid drug-induced alterations in the measurement of NMDA. All salts, amino acids, OPA (*o*-phthaldialdehyde), NAC (*N*-acetyl-L-cysteine) and other chemicals were purchased from Sigma Chemical Company. The Sep-Pak^® ^ODS C_18 _(octadecylsilyl-C_18_) cartridges were purchased from Waters Corporation, Milford, MA, USA.

### Tissue homogenization and purification of NMDA from other amino acids and amino compounds

Nerve cord, brain and muscle tissues were obtained from adults' animals and divided in five pools (each pool containing tissue from 20 animals). Samples were homogenized in 0.1 M TCA (trichloroacetic acid) in a ratio of 1:10 and centrifuged at 13,000 *g *for 5 minutes. The supernatants were purified to separate NMDA from other free amino acids and amino compounds. The sample purification was necessary to avoid interference of the NMDA peak by the peaks of other amino acids or amino compounds at HPLC. The principle of purification was based on the fact that free amino acids and amino compounds react with OPA (*o*-phthaldialdehyde) to give a complex "amino acid-OPA" which, if passed on an ODS-C_18 _Sep-Pak cartridge, binds strongly to the resin. NMDA instead, since it does not possess a primary amino group (NH_2_), does not react with OPA, therefore it does not form a complex with OPA and passes through the ODS-C_18 _column in purified form [9-12]. Briefly, the procedure is the following: 0.3 ml of TCA supernatant of tissue homogenate, as obtained above, is brought to pH 9.5 with 0.1 M NaOH and then mixed with 100 μl of 0.1 M sodium borate-boric acid buffer, pH 9.5 (to stabilize the pH), 20 μl of 1 M OPA and 2 μl of mercaptoethanol and incubated for 30 min at 37°C to complete the formation of the "OPA-amino acids" complex. After incubation, the mixture is brought to pH 8.2 with 0.1 M HCl and passed through an ODS-C_18 _cartridge containing about 800 mg of resin (the Sep-Pak had been previously regenerated by treatment with 10 ml of methanol and washed with 10 ml of distilled water). After the sample has been loaded on the cartridge, the eluent is collected. The cartridge is washed with 5 ml distilled water and this last eluent is pooled with the previous eluent and dried in a Petri dish, placed on a warm plate at 40–50°C and under aspiration in a hood until dried. The residue is dissolved in 0.3 ml distilled water and used for the HPLC determination of NMDA.

### Determination of NMDA by enzymatic HPLC method

**Figure 3:**
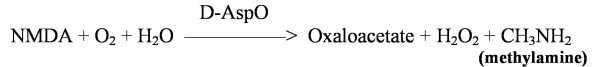
Oxidation reaction of NMDA by D-Aspartate oxidase and production of methylamine (CH_3_NH_2_).

This method is based on the measurement by HPLC of CH_3_NH_2 _(methylamine), which is generated by the oxidation of NMDA with D-aspartate oxidase (D-AspO) according to the reaction in Figure 3[9-11].

The CH_3_NH_2 _formed is then treated with OPA-mercaptoethanol to generate a fluorescent compound and analyzed by HPLC. The method is specific for NMDA since the purified beef kidney D-AspO oxidizes only D-Asp and NMDA [16-18] and only NMDA can generate CH_3_NH_2 _when NMDA is oxidized by the D-AspO [7,8]. Briefly, the procedure is as follows: 100 μl of purified sample is mixed with 50 μl of 0.2 M borate buffer, pH 8.2, and 5 μl of purified D-AspO (1–2 mg/ml; 200–300 U/ml) [16-18] and incubated at 37°C for 30 min. After incubation, 100 μl of 0.1 M sodium borate buffer, pH 10.0, and 5.0 μl of OPA-mercaptoethanol reagent (20 mg of OPA plus 40 μl of 2-mercaptoethanol in 2 ml of methanol) are added and mixed. After 2 min, 100 μl of this mixture is injected into an ODS-C_18 _Supelcosil column (μ ◂ 5 μm, 250 × 4.6 mm) connected to a Beckman-Gold HPLC system and to a fluorescence detector with excitation wavelength 330 nm and emission wavelength 450 nm. The column is eluted at 1.2 ml/min with a gradient consisting of 2 mobile phases, A and B. Solvent A consists of: 920 ml of double distilled water, 50 ml acetonitrile and 30 ml of 1.0 M citrate-phosphate buffer, pH 5.3 (final pH 5.6). Solvent B consists of: 900 ml acetonitrile and 100 ml double distilled water. The column is equilibrated with solvent A and then the following gradient program is carried out: 0–30% B over 3 min; 30–70% B over 11 min; 70–100% B over 4 min, 100% B for 4 min and return to 0% B in 1 min. The CH_3_-NH_2 _elutes as a sharp peak at the retention time of 11.8–12.0 min.

In the same way they are carried out a blank sample, a general standard and a general blank. The blank sample is prepared as the sample, but no D-AspO was added. The standard is prepared using 100 μl of NMDA at a concentration of 0.01 nmol/ml instead of the sample. The general blank is prepared using 100 μl of distilled water instead of the sample.

### D-Aspartate oxidase

D-Aspartate oxidase (D-AspO: EC 1.4.3.1) is an oxidative enzyme, which specifically catalyzed the oxidation of only the dicarboxylic amino acids in D-form (D-Asp, D-Glu and NMDA). However, it has been shown that the purified enzyme obtained from beef kidney oxidizes primarily D-Asp and NMDA and only minimally D-Glu (D-Glu is oxidized in a ratio of 2–3% compared to D-Asp or NMDA). On the other hand, the purified D-AspO obtained from the hepatopancreas of the mollusc *Octopus vulgaris *oxidizes only D-Asp and D-Glu, and only minimally NMDA (4–6% compared to D-Asp or D-Glu [16-18]). In this study we have used purified beef kidney D-aspartate oxidase obtained by recombinant expression in *E. coli *according to the described procedure [16].

### Biosynthesis of NMDA

Recently, we reported that the amphioxus nervous system contains considerable amounts of free D-aspartic acid [14]. Since this amino acid constitutes the precursor for the *in vivo *biosynthesis of NMDA [9-11], in the present study we have designed experiments to reveal if NMDA is biosynthesized from D-Asp in the amphioxus nervous system. To verify this, we used the described procedure [9] modified as follows: pools of nerve cord, cephalic vesicle/hindbrain and muscle, obtained from 10 animals were homogenized with 200 μl of 0.05 M phosphate buffer, pH 7.5, and centrifuged at 13,000 rpm for 10 min. Then, 100 μl of the supernatant was incubated with 10 μl of 0.2 M D-Asp (used as precursor for the biosynthesis of NMDA) and with 10 μl of 10 mM SAM (*S*-AdenosylMethionine, used as methyl group donor) for 2 h at 37°C. A blank sample was carried out in the same way as the sample, but without D-Asp. After incubation, the sample was mixed with 200 μl of 0.2 M TCA and centrifuged at 13,000 rpm for 5 min. The supernatant was neutralized with 0.1 M NaOH and was subjected to the purification of NMDA by using the above method for the determination of NMDA by HPLC. In the same assay conditions other amino acids (L-Asp, D-Glu, L-Glu, D-Ala, L-Ala, D-Ser, L-Ser and Gly) instead of D-Asp were used.

### Statistical analyses

Statistical analyses were performed using Statistical Pachage (Statsoft, 98^th ^Edition, 1977).

## Authors' contributions

SDA conceived the study and conducted experiments, analyzed the data and wrote the manuscript; GHF participated in the project design and helped to the final form of the manuscript; ET and GF provided technical assistance; JGF and ADA supported this work, coordinated the study and enhanced the final manuscript. All authors read and approved the manuscript and declare that they have no competing interests.
